# Supplementary material for: Nation-wide survey of oral care practice in Japanese intensive care units: A descriptive study
Source: PLoS One. 2024 Mar 29;19(3):e0301258. doi: 10.1371/journal.pone.0301258 (PMC10980190; doi:10.1371/journal.pone.0301258)
Supplement: S1 Fig — (DOCX) [file pone.0301258.s001.docx]

S1 Figure. Distribution of participants in Japan

Number of responses from all regions of Japan is presented; non-responses are in gray.
