# Supplementary material for: Nation-wide survey of oral care practice in Japanese intensive care units: A descriptive study
Source: PLoS One. 2024 Mar 29;19(3):e0301258. doi: 10.1371/journal.pone.0301258 (PMC10980190; doi:10.1371/journal.pone.0301258)
Supplement: S1 Table — (DOCX) [file pone.0301258.s002.docx]

S1 Table. Response to oral care frequency

| Survey item | | Equal intervals  (n=44) | Unequal intervals  (n=171) |
| --- | --- | --- | --- |
| Oral care frequency when performed at equal intervals (%) | |  | |
|  | <2 h | 0 (0.0) | NA |
|  | Every 4 h | 21 (47.7) | NA |
|  | Every 5 h | 1 (2.3) | NA |
|  | Every 6 h | 17 (38.6) | NA |
|  | Every 8 h | 4 (9.1) | NA |
|  | Every 12 h | 1 (2.3) | NA |
| Time of oral care when performed care at unequal intervals (%) | |  | |
|  | Daytime | NA | 1 (0.6) |
|  | Others | NA | 3 (1.8) |
|  | Morning, daytime | NA | 1 (0.6) |
|  | Morning, daytime, evening | NA | 116 (67.8) |
|  | Morning, daytime, evening, before sleep | NA | 21 (12.3) |
|  | Morning, daytime, before sleep | NA | 23 (13.5) |
|  | Morning, evening | NA | 3 (1.8) |
|  | Daytime, before sleep | NA | 1 (0.6) |
|  | Morning, evening, before sleep | NA | 2 (1.2) |
| Reason for performing oral care at unequal intervals (%) | |  | |
|  | A: Sleep is important during the night | NA | 9 (5.3) |
|  | B: Adaptation to time and frequency in daily life | NA | 55 (32.2) |
|  | C: Availability of work system and human resources | NA | 53 (31.0) |
|  | Others | NA | 3 (1.8) |
|  | A & B | NA | 11 (6.4) |
|  | A & C | NA | 10 (5.8) |
|  | B & C | NA | 18 (10.5) |
|  | A & B & C | NA | 12 (7.0) |

Abbreviations: NA, not applicable
