# Supplementary material for: Nation-wide survey of oral care practice in Japanese intensive care units: A descriptive study
Source: PLoS One. 2024 Mar 29;19(3):e0301258. doi: 10.1371/journal.pone.0301258 (PMC10980190; doi:10.1371/journal.pone.0301258)
Supplement: S2 Table — (DOCX) [file pone.0301258.s003.docx]

S2 Table. Response to non-brushing oral care

| Survey item | | Rinse only  (n=12) | Mouth swab only (n=74) | Combination of rinse and mouth swab  (n=33) |
| --- | --- | --- | --- | --- |
| Amount of liquid used for rinsing (median [IQR]) | | 7.5 [2.8, 20.0] | NA | 10.0 [5.0, 20.0] |
| Liquid mainly used for rinsing (%) | |  | | |
|  | Water | 6 (50.0) | NA | 15 (45.5) |
|  | Sterile water | 2 (16.7) | NA | 2 (6.1) |
|  | Mouthwash (over the counter or included in kit) | 3 (25.0) | NA | 14 (42.4) |
|  | Others | 1 (8.3) | NA | 2 (6.1) |
| Material mainly used for mouth swab (%) | |  | | |
|  | Gauze | NA | 5 (6.8) | 1 (3.0) |
|  | Foam swab | NA | 48 (64.9) | 25 (75.8) |
|  | Others | NA | 2 (2.7) | 1 (3.0) |
|  | Gauze & foam swab | NA | 17 (23.0) | 3 (9.1) |
|  | Gauze & others | NA | 0 (0.0) | 1 (3.0) |
|  | Gauze, foam swab, & others | NA | 2 (2.7) | 2 (6.1) |
| Liquid mainly used for mouth swab (%) | |  | | |
|  | Water | NA | 32 (43.2) | 16 (48.5) |
|  | Sterile water | NA | 2 (2.7) | 2 (6.1) |
|  | Mouthwash (over the counter or included in kit) | NA | 23 (31.1) | 12 (36.4) |
|  | Others | NA | 17 (23.0) | 3 (9.1) |

Abbreviations: IQR, interquartile range; NA, not applicable
