# Supplementary material for: Nation-wide survey of oral care practice in Japanese intensive care units: A descriptive study
Source: PLoS One. 2024 Mar 29;19(3):e0301258. doi: 10.1371/journal.pone.0301258 (PMC10980190; doi:10.1371/journal.pone.0301258)
Supplement: S3 Table — (DOCX) [file pone.0301258.s004.docx]

S3 Table. Response to moisturizing care

| Survey item | | Value  (N = 215 ICUs) |
| --- | --- | --- |
| Using something routinely for oral moisturization (%) | | 119 (55.3) |
| Product routinely used for oral moisturization (%)^a^ | |  |
|  | Oral balance^®^ | 31 (26.1) |
|  | Gel spray^®^ | 2 (1.7) |
|  | Refret care^®^ | 41 (34.5) |
|  | Others | 45 (37.8) |
| Apply antiseptic gel to the mouth (%) | | 32 (14.9) |
| Using routine lip moisturizer | | 101 (47.0) |
| Product routinely used for lip moisturization (%)^b^ | |  |
|  | Lip balm (over-the-counter) | 14 (13.9) |
|  | Exclusive products (over-the-counter) | 16 (15.8) |
|  | Artificial saliva | 1 (1.0) |
|  | Dedicated prescription drug | 70 (69.3) |
| Current oral care is effective in preventing VAP (median [IQR]) ^c^ | | 70.0 [51.3, 80.0] |

Abbreviations: VAP, ventilator-associated pneumonia; IQR, interquartile range

^a^Only those who responded that they use something routinely for oral moisturization (n=119).

^b^Only those who responded that they routinely use lip moisturizers (n=101).

^c^The maximum value was set at 100, and the respondents responded to the degree of the maximum value.
