# Supplementary material for: Nation-wide survey of oral care practice in Japanese intensive care units: A descriptive study
Source: PLoS One. 2024 Mar 29;19(3):e0301258. doi: 10.1371/journal.pone.0301258 (PMC10980190; doi:10.1371/journal.pone.0301258)
Supplement: S2 Text — (DOCX) [file pone.0301258.s006.docx]

S2 File. Questionnaire

**Responder**

1. Patient requiring ventilator is admitted

1. Yes
2. No ----> Questionnaire closed

2. Designation

1. Registered nurse
2. Nursing manager of each unit
3. Certified nurse specialist
4. Certified nurse

3. Nursing experience

1. <1 year
2. 1–5 years
3. 6–10 years
4. 11+ years

4. Nursing experience at current hospital

1. <1 year
2. 1–5 years
3. 6–10 years
4. 11+ years

5. Nursing experience in ICU

1. <1 year
2. 1–5 years
3. 6–10 years
4. 11+ years

6. Gender

1. Woman
2. Man

7. Total number of hospital beds

1. <200
2. 200–400
3. 401–600
4. 601–800
5. 801–1000
6. 1001–1200
7. 1201–1400

8. Number of ICU beds

1. Select a value （1–60)

9. University hospital

1. Yes
2. No

10. ICU in emergency department

1. Yes
2. No

11. Staffing of doctor in the ICU

1. Intensivist is the attending physician
2. Intensivists are not attending physicians; however, all patients admitted to the ICU are seen by an intensivist
3. Intensivists are involved in patient care only when the attending physician requests to see them
4. No intensivists

12. Number of day-shift weekday working nurses

1. 【 　】 (Please enter the number)

13. Number of night-shift weekday working nurses

1. 【　 】 (Please enter the number)

14. Standard or common oral care procedures exist across the unit.

（No matter if manuals are available or unavailable）

1. Yes ----> A-1
2. No ----> Survey closed

**A. Evaluation**

A-1. Oral evaluation is performed using a scale immediately upon ICU admission

1. Yes ----> A-2
2. No ----> A-3

A-2. Type of scale used

1. Oral Assessment Guide（OAG）
2. Revised Oral Assessment Guide（ROAG）
3. Oral Health Assessment Tool (OHAT-J)
4. Others

A-3. Oral evaluations are routinely performed using a scale

1. Yes ----> A-4
2. No ----> B-1

A-4. Type of scale used

1. Oral Assessment Guide（OAG）
2. Revised Oral Assessment Guide（ROAG）
3. Oral Health Assessment Tool (OHAT-J)
4. Others

**B. Frequency**

B-1. Oral care frequency

1. 【　　】 time/day (0–24)

B-2. Perform oral care at equal intervals

1. Yes (X h interval） ----> B-3
2. No ----> B-4

B-3. Oral care frequency when performed at equal intervals

1. <2 h
2. Every 3 h
3. Every 4 h
4. Every 5 h
5. Every 6 h
6. Every 7 h
7. Every 8 h
8. Every 12 h
9. Every 24 h ----> C-1

B-4. Timing of oral care when performed at unequal intervals （multiple options available）

1. Morning
2. Daytime
3. Evening
4. Before sleep
5. Others

B-5. Reason for performing oral care at unequal intervals (multiple options available)

1. Sleep is important during the night
2. Adaptation to time and frequency in daily life
3. Availability of work system and human resources
4. Others

**C. Methods**

C-1. Adjusting cuff pressure before oral care

1. Yes ----> C-2
2. No ----> D-1

C-2. Pressure for cuff adjustment【　　】 cmH_2_O

**D. Toothbrushing for oral care**

D-1. Using a toothbrush for daily oral care.

1. Using for every oral care ----> D-3（skip to F after answering D）
2. Combined with non-using oral care ----> D-2
3. Not used ----> E-1

D-2. Toothbrushing frequency

1. 【　】time(s)/day

D-3. Toothbrush type

1. Adult
2. Child
3. Electric toothbrush

D-4. Single-use disposable toothbrush

1. Yes
2. No

D-5. Using toothpaste

1. Yes
2. No

D-6. Rinsing after brushing

1. Yes
2. No --->E-1 （skip to F if 1 was answered to D-1）

D-7. Amount of liquid used for rinsing after brushing

1. 【　　】 ml/time

D-8. Liquid mainly used after brushing

1. Water
2. Normal saline
3. Sterile water
4. Mouthwash (over-the-counter or included in kit)
5. Povidone-iodine
6. Hydrogen peroxide solution
7. Chlorhexidine
8. Others

**E. Non-brushing oral care** (Only if 2 or 3 was answered to D-1 )

E-1. Rinsing or mouth swab for non-brushing oral care

1. Only rinsing ----> E-3 + E-4 ----> F-1
2. Only mouth swab ---> E-5 + E-6 ----> F-1
3. Both rinsing and mouth swab ----> E-3

E-2. Amount of liquid used for rinsing

1. 【　　】mL

E-3. Liquid mainly used for rinsing

1. Water
2. Normal saline
3. Sterile water
4. Mouthwash (over-the-counter or included in kit)
5. Povidone-iodine
6. Hydrogen peroxide solution
7. Chlorhexidine
8. Others

E-4. Material mainly used for mouth swab (multiple options available)

1. Gauze
2. Foam swab
3. Others

E-5. Liquid mainly used for mouth swab

1. Water
2. Normal saline
3. Sterile water
4. Mouthwash (over-the-counter or included in kit)
5. Povidone-iodine
6. Hydrogen peroxide solution
7. Chlorhexidine
8. Others

**F. Moisturizing care**

F-1. Using something routinely for oral moisturization

1. Yes
2. No ----> F-3

F-2. Product routinely used for oral moisturization

1. Oral balance^®^
2. Gel spray^®^
3. Refret care^®^
4. Others

F-3. Applying antiseptic gel to the mouth

1. Yes
2. No

F-4. Using routine lip moisturizer

1. Yes
2. No ----> G-1

F-5. Product routine used for lip moisturization

1. Lip balm (over-the-counter)
2. Exclusive products (over-the-counter)
3. Artificial saliva
4. Dedicated prescription drug

**G. Care equipment**

G-1. Using the special oral care kits.

1. Yes
2. No

G-2. Patients or family members are responsible for purchasing the special oral care kits.

1. Yes
2. No

**H. Outcomes**

H-1. Degree to which current oral care is effective in preventing VAP

1. 【　　】（VAS：1–100)

H-2. Monitoring for ventilator-associated infections such as VAP and VAE.

1. Yes
2. No
