# Supplementary material for: Nation-wide survey of oral care practice in Japanese intensive care units: A descriptive study
Source: PLoS One. 2024 Mar 29;19(3):e0301258. doi: 10.1371/journal.pone.0301258 (PMC10980190; doi:10.1371/journal.pone.0301258)
Supplement: Attachment — Submitted filename: Response_to_reviewers.docx [file pone.0301258.s007.docx]

**Response to Editor and Reviewers**

***Journal Requirements***

***Comment 1***

*Please ensure that your manuscript meets PLOS ONE's style requirements, including those for file naming. The PLOS ONE style templates can be found at*
[*https://journals.plos.org/plosone/s/file?id=wjVg/PLOSOne_formatting_sample_main_body.pdf*](https://journals.plos.org/plosone/s/file?id=wjVg/PLOSOne_formatting_sample_main_body.pdf) *and*
[*https://journals.plos.org/plosone/s/file?id=ba62/PLOSOne_formatting_sample_title_authors_affiliations.pdf*](https://journals.plos.org/plosone/s/file?id=ba62/PLOSOne_formatting_sample_title_authors_affiliations.pdf)

**Response**

Thank you for the comments. We have revised the manuscript and ensured that it conforms with the *PLOS ONE* style.

***Comment 2***

*Please provide additional details regarding participant consent. In the ethics statement in the Methods and online submission information, please ensure that you have specified what type you obtained (for instance, written or verbal, and if verbal, how it was documented and witnessed). If your study included minors, state whether you obtained consent from parents or guardians. If the need for consent was waived by the ethics committee, please include this information.*

**Response**

Thank you for pointing this out. In this study, the Ethics Committee had approved a consent form waiver. We have described the details of which as follows:

Page 5, Line 104 - L109

We sent the information of this study with a QR code for the SurveyMonkey questionnaire by postal mail. The first page of the survey form included study information and a section where participants could confirm their consent to take part. To indicate their willingness to participate, individuals submitted their responses and marked a checkbox next to the consent statement. The Ethics Committee reviewed these checkboxes and approved the consent form, considering them as a valid expression of consent.

***Comment 3***

*Thank you for stating the following in the Competing Interests:
 "I have read the journal's policy and the authors of this manuscript have the following competing interests: Dr. Unoki has received research grants from Alcare Co. Ltd. The remaining authors report no conflicts of interest. "*

*We note that one or more of the authors have an affiliation to the commercial funders of this research study : Alcare Co. Ltd.*

*a. Please provide an amended Funding Statement declaring this commercial affiliation, as well as a statement regarding the Role of Funders in your study. If the funding organization did not play a role in the study design, data collection and analysis, decision to publish, or preparation of the manuscript and only provided financial support in the form of authors' salaries and/or research materials, please review your statements relating to the author contributions, and ensure you have specifically and accurately indicated the role(s) that these authors had in your study. You can update author roles in the Author Contributions section of the online submission form.*

*Please also include the following statement within your amended Funding Statement.
“The funder provided support in the form of salaries for authors [insert relevant initials], but did not have any additional role in the study design, data collection and analysis, decision to publish, or preparation of the manuscript. The specific roles of these authors are articulated in the ‘author contributions’ section.”
If your commercial affiliation did play a role in your study, please state and explain this role within your updated Funding Statement.*

*b. Please also provide an updated Competing Interests Statement declaring this commercial affiliation along with any other relevant declarations relating to employment, consultancy, patents, products in development, or marketed products, etc.*

*Within your Competing Interests Statement, please confirm that this commercial affiliation does not alter your adherence to all PLOS ONE policies on sharing data and materials by including the following statement: "This does not alter our adherence to PLOS ONE policies on sharing data and materials.” (as detailed online in our guide for authors* [*http://journals.plos.org/plosone/s/competing-interests*](http://journals.plos.org/plosone/s/competing-interests)*). If this adherence statement is not accurate and there are restrictions on sharing of data and/or materials, please state these. Please note that we cannot proceed with consideration of your article until this information has been declared.*

**Response**

Thank you for your comment. We received our research funding from Alcare Co., but the company did not pay salaries to any authors and is not fully involved in the research. Therefore, we sent an enquiry e-mail to Nicholas Wrigley, Publications Assistant and received a confirmation regarding this information.

The company did not play a role in study design, data collection and analysis, publication decisions, or manuscript preparation. None of the remaining authors have an affiliation with the funders of this research study.

***Additional Editor Comments***

*One reviewer noted some concerns regarding the generalisability of the study. This point should be addressed in more depth in the discussion.*

*In particular, the importance of the study's publication in an international scientific journal should be mentioned in the discussion. The current situation in Japan is discussed in depth and is of great significance here. However, please add what implications the results of the Japanese study have for practice in other countries, such as the USA and the European bloc.*

*These opinions and changes will be passed on to a different Reviewwe than the Reviewer who made the decision to Reject.*

*Please add an additional note regarding the international generalisability of this research.*

**Response**

Thank you for your comments. We revised our manuscript following your comments. We additionally describe the importance of this study for international science and generalizability in the discussion section.

Page 13 - 14, Line 230 - L 237

The questionnaire survey may not provide a precise reflection of actual oral care practices. Thus, a one-point prevalence study could be instrumental in elucidating the genuine state of oral care. Moreover, the findings from this study revealed disparities between Japanese oral care methods and those from other countries. These results also underscore the influence of traditional cultures and lifestyles on varying oral care practices in different regions and countries. Consequently, the absence of a universally accepted standard for oral care is evident, and there is a need for a clearer understanding of the impact of various oral care approaches in preventing VAP. This suggests the necessity for a randomized controlled trial to establish concrete evidence regarding the efficacy of different oral care methods.

***Reviewers’ comments***

**Reviewer #1**

***Comment***

*This was a cross-sectional (survey) study evaluating the practices of oral hygeine in criticaly ill patients in Japan.*

*I would like to thank and commend the authors for a job very well done.*

*The artice is well written, the topic is of great importance, and the manuscript incudes all the structural components of an article.*

*I have no further comments, and recommend acceptance.*

**Response**

We greatly appreciate the insightful comments on our manuscript.

**Reviewer #2**

*Thank you for the invitation to review this manuscript.*

*The author present the results of an online survey of oral nursing care in Japanese ICUs. Based upon the limited response they received, the authors believe that practicies in Japan differ from those reported in the US and Europe.*

***Comment 1***

*This survey does not meet the first PLOS ONE criteria for publication, namely, "the results of primary scientific research.*

**Response**

Thank you for your valuable review and comments. We have carefully reviewed the *PLOS ONE* publication criteria and believe our study constitutes primary scientific research. In support of this assertion, we followed a rigorous academic methodology in line with the STROBE statement, conducting a nationwide study in Japan that encompassed hospitals all over Japan.

Although guidelines for oral care have been published, this is one example of how oral care varies from culture to culture. We believe this study is valuable for creating a global standard of oral care.

***Comment 2***

*There is an absense of evidence that oral care in orally / nasally intubated patients has any patient-centred measureable outcome benefit. As a comfort measure however, it has obvious value.*

**Response**

Thank you for your insightful comment. As you indicated, we have revised the discussion in the manuscript as follows, considering the importance of assessing the patient comfort with the assessment tool.

Page 12, Line 193 - L 198

However, our findings may be insufficient according to international consensus. The British Association of Critical Care Nurses (BACCN) provided a consensus statement for oral care in 2021 and recommended that oral condition should be assessed with a standardized tool within 6 h of ICU admission and repeated within 12 h at least [9]. The oral assessment tools not only assess the oral condition but also the comfort of the patient. Thus, using the assessment tool to ensure validity may improve the quality of life for patients during hospitalization.

***Comment 3***

*What does it change knowing that that there are somewhat different ICU nursing practices in different coutries in regard to oral care in intubated patients?*

**Response**

Thank you for your important comment. We should have described the generalizability of the study sufficiently. We have revised the research implication in the manuscript follows:

Page 13 - 14, Line 230 - L 237

The questionnaire survey may not provide a precise reflection of actual oral care practices. Thus, a one-point prevalence study could be instrumental in elucidating the genuine state of oral care. Moreover, the findings from this study revealed disparities between Japanese oral care methods and those from other countries. These results also underscore the influence of traditional cultures and lifestyles on varying oral care practices in different regions and countries. Consequently, the absence of a universally accepted standard for oral care is evident, and there is a need for a clearer understanding of the impact of various oral care approaches in preventing VAP. This suggests the necessity for a randomized controlled trial to establish concrete evidence regarding the efficacy of different oral care methods.
