# Supplementary material for: Nation-wide survey of oral care practice in Japanese intensive care units: A descriptive study
Source: PLoS One. 2024 Mar 29;19(3):e0301258. doi: 10.1371/journal.pone.0301258 (PMC10980190; doi:10.1371/journal.pone.0301258)
Supplement: Attachment — Submitted filename: Responses_to_reviewers.docx [file pone.0301258.s008.docx]

Response to Reviewers

Dear Editor and Reviewers

Thank you very much for reviewing our manuscript and offering valuable advice. We have addressed your comments with point-by-point responses and revised the manuscript accordingly.

***Comments from Academic Editor***

・Please correct the points raised by the reviewer who judged it as Major Revision. please refer to the opinions of the reviewer who judged it as Reject if they can be queried.

・Please make every effort to refer to the opinions of Reviewers who have been judged as Reject if you can.

・Please note that the Reviewer has stated that he/she will leave it to the Editor to decide on the significance of submitting to PLOS ONE for research conducted in Japan.

　We believe that there is no problem with the Editor's decision on this point. Please reply to this matter by stating that the "Editor decided that there is no problem".

**Response**

We appreciate your helpful comment on our manuscript. We have revised our manuscript, addressing the major revision points raised by the reviewers and incorporating suggestions from those who recommended rejection to the best of our ability.

***Comments from Reviewer #3:***

**General comments**

The authors reported the frequency and methods of oral care in patients admitted to the intensive care units (ICU) in Japan. They found that the most common frequency of oral care was thrice a day (68.8%), and most ICUs provided the care at unequal intervals in a day.

They conclude that oral care practices in Japan differ from those in other countries, especially in frequency and methods.

This article's topic may be valuable for the field of intensive care in terms of preventing ventilator-associated pneumonia.

However, from the reviewer's perspective, this article is just a report, not original research. In conclusion, the authors claimed that oral care practices in Japan differs from those in other countries without direct data collection from those countries. Their original data, which is only collected from Japanese ICUs, does not support their conclusion.

These data are valuable to publicize elsewhere, but this is not suitable for publicizing as an original research article, at least in the current form.

**Response**

We appreciate your helpful comments on our manuscript. We have revised our manuscript based on your suggestions. In addition, the editor has informed us about the general comments of reviewer #3, stating that “Editor decided that there is no problem.”

**Minor comments**

**Comment**

P4L74: Is it true the authors included all ICUs in Japan? Please describe the definition of ICU, and what list the authors used to pick up ICUs in hospitals in Japan.

**Response**

Thank you for your insightful review of our paper. We regret that our manuscript contained inaccuracies and lacked a detailed description of the sample and setting section. Therefore, we have added a detailed description of the sample and setting section and revised the abstract as follows:

Page 2, Line 26 – L 30

Thus, this study aimed to determine the overall oral care practices, including those for intubated patients, in Japanese intensive care units. This study included all Japanese intensive care units meeting the authorities' standard set criteria, with a minimum of 0.5 nurses per patient at all times and admission of adult patients requiring mechanical ventilation. An online survey was used to collect data.

Page 4, Line 77 – L 87

Japanese ICUs that meet the standards set by the Japanese Ministry of Health, Labour and Welfare (MHLW) are mainly classified into two types: general ICUs and ICUs specializing in emergency medical care. The former includes four types of ICUs with a minimum of 0.5 nurses per patient at all times, while the latter includes ICUs with at least 0.5 nurses per patient at all times and ICUs with at least 0.25 nurses per patient at all times. In Japan, the ICUs with at least 0.5 nurses per patient at all times are major facilities. Thus, in this study inclusion criteria were as follows: (1) ICUs approved by the MHLW with at least 0.5 nurses per patient ratio at all times and (2) ICUs admitting patients requiring mechanical ventilation. Pediatric ICUs were excluded.

Postal mails were sent to nurse directors at all hospitals with eligible ICUs requesting their participation in the survey and referral of one nurse with actual oral care practice in the ICU as the survey respondent. Thus, one response per ICU was received.

**Comment**

Table 1: Are the characteristics of respondents (e.g., gender, designation, nursing experience, etc.) necessary? Do these characteristics affect something in the results?

**Response**

Thank you for pointing this out. We have removed gender, designation, nursing experience, nursing experience at current hospital, and nursing experience in ICU from the Results section and Table 1 based on your suggestion. In addition, we have revised the title of Table 1, please see Table 1.

Page 6, Line 128 – L 131

Characteristics of participating ICUs are listed in Table 1. Regarding participating hospitals, most respondent hospitals had approximately 401–600 beds. In addition, the respondent hospitals had a median of 8 (IQR 6–12) and 7.5 (IQR 6–10) ICU beds for the standardized and non-standardized groups, respectively.

Page 6, Line 134

Table 1. Characteristics of participating ICUs

***Comments from Reviewer #4:***

Thank you for allowing me to review "Nation-Wide Survey of Oral Care Practice in Japanese Intensive Care Unit : a Descriptive Study" by Kuribara, Unoki et al.

The authors performed survey of oral care for intubated patients in Japanese ICU.

This is an interesting study. However, it is a difficult question whether a study that merely reports the results of this Japan-only survey is appropriate for an international journal. I leave it to the editorial board to decide, but a letter might be more appropriate.

My suggestions are as follows.

**Response**

We appreciate your helpful comments. Please see our responses to each suggestion below:

**Abstract**

**Comment**

“A total of 609 hospitals and 717 ICUs nationwide participated; among these, 215 (30.0%) and 32 (13%) reported standardized and non-standardized oral care, respectively”

215/717=30%

However, 32/717=4.4%

Please confirm.

I think that responses were collected from 247 ICUs.

This information should be included in the abstract.

**Response**

Thank you for pointing this out. We are very sorry. The denominator was incorrect for some of the values during calculation. In this sentence, we set “247” as the denominator. Therefore, we have reviewed and revised the values in the abstract and main text to accurately reflect the correct values.

Page 2, Line 32 – L 36

A total of 609 hospitals and 717 intensive care units nationwide participated; among these, responses were collected from 247 intensive care units (34.4%). Of these, 215 (87.0%) and 32 (13.0%) reported standardized and non-standardized oral care, respectively. Subsequently, the data from 215 intensive care units that provided standardized oral care were analyzed in detail.

**Comment**

“Regarding oral care methods, 96 (38.9%) respondents used only a toothbrush, while 116 (46.9%) used both a toothbrush and a non-brushing method.”

How much is the denominator?

It is confusing.

**Response**

Thank you for your insightful review of our paper. We set “215” as the denominator; however, the denominator was precisely unclear in our abstract. Therefore, we revised our abstract to clearly reflect the denominator as follows:

Page 2, Line 32 – L 36

A total of 609 hospitals and 717 intensive care units nationwide participated; among these, responses were collected from 247 intensive care units (34.4%). Of these, 215 (87.0%) and 32 (13.0%) reported standardized and non-standardized oral care, respectively. Subsequently, the data from 215 intensive care units that provided standardized oral care were analyzed in detail.

**Comment**
“differed from those in other countries.”

How different?

**Response**

Thank you for your comment. We revised our abstract based on your comment to make this point clear.

Page 2, Line 39 – L 43

Our study revealed differences in current oral care practices in Japan compared to other countries, particularly the practice of brushing three times a day at unequal intervals and the common use of brushing for oral care. Oral care practices in intensive care units vary worldwide; therefore, more evidence is needed to consider the cultural influences on oral care methods in each country.

**Introduction**

**Comment**

“Providing oral care for critically ill patients is crucial for their comfort”

Please provide references.

**Response**

Thank you for your comment. We provided additional references as follows:

Page 3, Line 50 – L 52

Providing oral care for critically ill patients is crucial for their comfort, improvement of thirst [1,2], and preventing ventilator-associated pneumonia (VAP). Oral care helps improve oral health and reduce inflammation [3,4].

References

Kalfon P, Mimoz O, Auquier P, Loundou A, Gauzit R, Lepape A, et al. Development and validation of a questionnaire for quantitative assessment of perceived discomforts in critically ill patients. Intensive Care Med. 2010;36:1751-1758. doi: 10.1007/s00134-010-1902-9.

Doi S, Nakanishi N, Kawahara Y, Nakayama S. Impact of oral care on thirst perception and dry mouth assessments in intensive care patients: An observational study. Intensive Crit Care Nurs. 2021;66:103073. doi: 10.1016/j.iccn.2021.103073.

**Comment**

“However, a standardized protocol for oral care is yet to be established, with each hospital following its own protocol.”

This sentence should be moved to the previous paragraph.

**Response**

Thank you for your insightful review of our paper. We agree with your suggestion. Thus, we have moved the corresponding text to the previous paragraph in the Introduction section.

Page 3, Line 52 – L 57

Oral care for intubated patients can reduce the incidence of VAP, ventilator days, and length of intensive care unit (ICU) stay [5]. Nurses in many countries recognize the importance of oral care for such patients [6, 7]. However, a standardized protocol for oral care has yet to be established, with each hospital following its own protocol.

Several countries have conducted studies on the actual situation of oral care in ICUs in the past decade [8, 9].

**Materials and Methods**

**Data analysis**

**Comment**

“The responses to the questionnaire were analyzed by calculating the frequency and percentage of each 97 response.”

What is the difference between the frequency and percentage?

**Response**

Thank you for your comment. We have revised our manuscript based on the reviewer's comment as follows:

Page 5, Line 104 – L 107

The responses to the questionnaire were analyzed using descriptive analysis. Categorical variables were calculated as the percentage of each response. For continuous variables, the median and interquartile range (IQR) were calculated for non-normal distributions, and the mean and standard deviation (SD) for normal distributions.

**Results**

**Comment**

Participant characteristics

I think information such as gender, experience and designation of the responding nurses is unnecessary for the results and Table1.

**Response**

Thank you for your comment. Based on your suggestions, we have removed gender, designation, nursing experience, nursing experience at current hospital, and nursing experience in ICU from the Results section and Table 1. In addition, we have revised the name of Table 1, please see Table 1.

Page 6, Line 128 – L 131

Characteristics of participating ICUs are listed in Table 1. Regarding participating hospitals, most respondent hospitals had approximately 401–600 beds. In addition, the respondent hospitals had a median of 8 (IQR 6–12) and 7.5 (IQR 6–10) ICU beds for the standardized and non-standardized groups, respectively.

Page 6, Line 134

Table 1. Characteristics of participating ICUs

**Comment**

Table 1. Participant characteristics

“Intensivist is the attending physician”, “Intensivists are not attending physicians; however, all patients admitted to the ICU are seen by an intensivist”, “Intensivists are involved in patient care only when the attending physician requests to see them”

The explanation is a bit redundant; why not state closed ICU (J Intensive Care. 2018 Sep 3:6:57.), semi closed ICU, open ICU, etc., and put the explanation below the table?

**Response**

Thank you for your insightful comment. We have reviewed the reference reviewer provided and we agree with your suggestions. Thus, we revised Table 1 based on your suggestions, please see Table 1.

Page 7, Line 136 – L139

^a^Intensivist is the attending physician.

^b^Intensivists are not attending physicians; however, all patients admitted to the ICU are visited by an intensivist.

^c^Intensivists are involved in patient care only when the attending physician requests to see them.

**Comment**

Table 2. Response to oral condition assessment

“Frequency”

The number of the hospital (%)?

**Response**

Thank you for pointing this out. The column heading “Frequency” was unclear, and we have revised it to “Value (n = 215 ICUs)”. Please see Table 2.

**Comment**

Table 3. Response to toothbrushing and other methods of oral care

Same as above.

**Response**

Thank you for pointing this out. The column heading “Frequency” was unclear, and we have revised it to “Value (n = 212 ICUs)”. Please see Table 3.

**Discussion**

**Comment**

“Furthermore, the differences and similarities between Japanese ICUs and those in other countries were highlighted.”

The differences between Japanese ICUs and ICUs in other countries was described, but what are the similarities?

**Response**

We appreciate your helpful comment. There are similarities in the insufficient repeated use of assessment tools after ICU admission between Japan and other countries compared to the international consensus. We have added to the discussion section to make this point clear.

Page 11, Line 199 - 204

A study revealed that 35% of nurses in the United Kingdom used oral assessment tools during ICU admission [14], and another study reported that most nurses in the United States did not use oral assessment tools [8]. The current state of oral care in Japan, as found in our study, shows the difference in the rate of use of oral assessment tools compared to that reported in the United Kingdom and the United States. However, there may be insufficient repeated use of assessment tools compared to international consensus, the same as the United Kingdom and the United States.

**Comment**

“However, our findings may be insufficient according to international consensus.”

It is difficult to understand the meaning of this sentence.

Do authors mean “However, the current state of oral care in Japan, as found in our study, may be insufficient according to the international consensus.”

**Response**

Thank you for your helpful comment. We have revised our manuscript in response to the reviewer’s suggestion as follows:

Page 11, Line 199 - 204

A study revealed that 35% of nurses in the United Kingdom used oral assessment tools during ICU admission [14], and another study reported that most nurses in the United States did not use oral assessment tools [8]. The current state of oral care in Japan, as found in our study, shows the difference in the rate of use of oral assessment tools compared to that reported in the United Kingdom and the United States. However, there may be insufficient repeated use of assessment tools compared to international consensus, the same as the United Kingdom and the United States.
