# Supplementary material for: Nation-wide survey of oral care practice in Japanese intensive care units: A descriptive study
Source: PLoS One. 2024 Mar 29;19(3):e0301258. doi: 10.1371/journal.pone.0301258 (PMC10980190; doi:10.1371/journal.pone.0301258)
Supplement: Attachment — Submitted filename: Response to reviewers.docx [file pone.0301258.s009.docx]

Response to Reviewers

***Dear Editor and Reviewers:***

Thank you very much for reviewing our manuscript and offering valuable advice. We have addressed your comments with point-by-point responses and revised the manuscript accordingly.

***Comments from Academic Editor***

Thank you for resubmitting the manuscript.

Reviewer raise some concerns, and I please revise accrding the reviewers comments.

Especially,

・"The first paragraph of a discussion should generally describe what was found by the study. "

If you cannot fully responce the comment by reviewer, you can add the limitation of your study.

**Response**

Thank you for the valuable advice and recommendations. We have revised our manuscript according to each comment. Our point-by-point responses are given below.

***Comments from Reviewer #4:***

Thank you for allowing me to review "Nation-Wide Survey of Oral Care Practice in Japanese Intensive Care Unit : a Descriptive Study" by Kuribara, Unoki et al.
The authors addressed the reviewer’s comments.
As stated in the previous review, it is difficult to see whether this study is appropriate to be published as a full paper. I leave it to the editorial board to decide, but a letter might be more appropriate.

My comments are as follows.

**Response**

We appreciate your helpful comments regarding our manuscript. We have revised our manuscript according to your comments and provided point-by-point responses below.

**Comment**
Abstract
“Our study revealed differences in current oral care practices in Japan compared to other countries, particularly the practice of brushing three times a day at unequal intervals and the common use of brushing for oral care.”
This study only reports the results of a questionnaire on the current situation in Japan and does not identify any differences between Japan and other countries. This statement is therefore inappropriate and needs to be corrected.
Also, my question, ' How different?' is not answered.

**Response**

Thank you for your valuable insights. We sincerely apologize for our inadequate responses and revisions in the previous round. Although this study has clarified the differences between ICUs in Japan and those in other countries through its discussion, as you point out, this study only reports the results of a questionnaire on the current situation in Japan and does not identify any differences between Japan and other countries through its findings. Therefore, we have modified the aim and conclusions in the Abstract and main text. We have also made some changes, visible in red, throughout the manuscript; these revisions emphasize that the differences being discussed are specific to ICUs in Japan and those in other countries.

Page 2, Lines 27–29

Thus, this study aimed to determine the overall oral care practices, including those for intubated patients, in Japanese intensive care units. We also discuss the differences in oral care methods between Japanese ICUs and ICUs in other countries.

Page 2, Lines 41–44

The findings of our study reveal current oral care practices in ICUs in Japan. In particular, most ICUs provide oral care three times a day at unequal intervals, and almost all use toothbrushes as a common tool for oral care. The results suggest that some oral care practices in Japanese ICUs differ from those in ICUs in other countries.

Page 3, Lines 67–70

Therefore, this study aimed to determine the overall oral care practices, including those for intubated patients, in ICUs throughout Japan. We also discuss the differences in oral care methods between Japanese ICUs and ICUs in other countries.

Page 13, Lines 253–256

In summary, most ICUs in Japan provide oral care three times a day at unequal intervals, and almost all use toothbrushes as a common tool for oral care. The results suggest that some oral care practices in Japanese ICUs differ from those in ICUs in other countries. Future studies should focus on the efficacy of different oral care methods to provide more evidence.

**Comment**
Discussion
“Furthermore, the differences and similarities between Japanese ICUs and those in other countries were highlighted.”
The authors mentioned similarity, but I still don't think it is an appropriate sentence to include in the first paragraph of a discussion. The first paragraph of a discussion should generally describe what was found by the study. For the readers, the difference and similarity are not highlighted at this point. It should be explained in detail in the paragraphs to come.

**Response**

Thank you for your valuable comments. We agree with your suggestion and have modified the first paragraph of the Discussion to show the main findings of the study.

Page 11, Line 191–195

The findings of this study revealed current oral care practices in ICUs in Japan. In particular, most ICUs provided oral care three times a day at unequal intervals, mainly in the morning, daytime, and evening, and almost all used toothbrushes as a common tool for oral care, with water being the predominant choice for rinsing the mouth after brushing. Approximately half of ICUs used oral assessment tools or provided moisturizing care in addition to toothbrushes.
